# Highlights From the American Heart Association's EPI|LIFESTYLE 2019 Scientific Sessions

**DOI:** 10.1161/JAHA.119.012925

**Published:** 2019-06-01

**Authors:** Alvaro Alonso, Madison D. Anderson, Michael P. Bancks, Sherry‐Ann Brown, Melissa C. Caughey, Alex R. Chang, Erin Delker, Kathryn Foti, Véronique Gingras, Michael G. Nanna, Alexander C. Razavi, Jewel Scott, Elizabeth Selvin, Catherine Tcheandjieu, Alvin G. Thomas, Ruth‐Alma N. Turkson‐Ocran, Allison Webel, Deborah R. Young, Bailey M. DeBarmore

**Affiliations:** ^1^ Department of Epidemiology Rollins School of Public Health Emory University Atlanta GA; ^2^ Division of Epidemiology and Community Health University of Minnesota, School of Public Health Minneapolis MN; ^3^ Division of Public Health Sciences Department of Epidemiology & Prevention Wake Forest School of Medicine Winston‐Salem NC; ^4^ Department of Cardiovascular Diseases Mayo Clinic Rochester MN; ^5^ Division of Cardiology University of North Carolina Chapel Hill NC; ^6^ Kidney Health Research Institute Geisinger Health System Danville PA; ^7^ Joint Doctoral Program Public Health Epidemiology San Diego State University and University of California at San Diego San Diego CA; ^8^ Department of Epidemiology Johns Hopkins Bloomberg School of Public Health Baltimore MD; ^9^ Division of Chronic Disease Research Across the Lifecourse Department of Population Medicine Harvard Medical School and Harvard Pilgrim Health Care Institute Boston MA; ^10^ Duke Clinical Research Institute Durham NC; ^11^ Division of Cardiology Duke University School of Medicine Durham NC; ^12^ Department of Medicine Tulane University School of Medicine New Orleans LA; ^13^ Department of Epidemiology Tulane University School of Public Health and Tropical Medicine New Orleans LA; ^14^ Duke University School of Nursing Durham NC; ^15^ Welch Center for Prevention, Epidemiology, and Clinical Research Johns Hopkins University Baltimore MD; ^16^ Division of General Internal Medicine Department of Medicine Johns Hopkins School of Medicine Baltimore MD; ^17^ Department of Pediatric Cardiology Stanford Cardiovascular Institute Stanford School of Medicine Stanford CA; ^18^ Department of Epidemiology Gillings School of Global Public Health University of North Carolina at Chapel Hill NC; ^19^ Johns Hopkins University School of Nursing Baltimore MD; ^20^ Frances Payne Bolton School of Nursing Case Western Reserve University Cleveland OH; ^21^ Department of Research and Evaluation Kaiser Permanente Southern California Pasadena CA

**Keywords:** cardiovascular disease, epidemiology, genetic epidemiology, lifestyle, prevention, Epidemiology, Lifestyle

This year's American Heart Association (AHA) EPI|Lifestyle Scientific Sessions were held March 5 to 8, 2019 in Houston, Texas, a combined meeting of the Council on Epidemiology and Prevention and the Council on Lifestyle and Cardiometabolic Health. The meeting focused on the influence of omics, traditional and emerging risk factors, social determinants, and lifestyle behaviors on cardiovascular health (Box 1).^1^

Box 1 **Learning Objectives for AHA EPI|Lifestyle 2019 Scientific Sessions**

**Table:** 

*Modifiable risk factors*: Recognize opportunities to address modifiable risk factors for cardiovascular disease to help meet AHA components of ideal cardiovascular health
*Health disparities*: Discuss the causes and health consequences of continuing disparities in cardiovascular disease risk factors among certain racial/ethnic, age, sex, and socioeconomic groups
*Built environment*: Describe the impact of the built environment on the health of populations and identify research and preventative opportunities to improve such environments
*Primordial and primary prevention*: Discuss current guidelines and research needs related to primordial and primary cardiovascular disease prevention in children and adolescents
*Precision medicine*: Explain the concept of precision medicine and identify opportunities and applications for precision medicine in cardiovascular disease prevention and treatment
*Early career*: Support early‐career researchers and clinicians who are pursuing career opportunities and pathways in epidemiology and prevention of cardiovascular disease

Source: AHA EPI|Lifestyle Programming Page, Final Program.^1^

This year's conference hosted over 300 poster presentations, including 72 well‐attended moderated poster sessions on topics ranging from novel cardiovascular biomarkers and clinical trials to nutritional epidemiology and social determinants of health. The annual National Heart Lung and Blood Institute trainee session featured 15 oral abstracts presented by trainees from 10 institutions and 6 moderated posters. These early career researchers presented new insights on genes, behavior, and environment from several major National Institutes of Health–funded cohort studies including the ARIC (Atherosclerosis Risk in Communities) study, Framingham Heart Study, Jackson Heart Study, MESA (Multi‐Ethnic Study of Atherosclerosis), and the HCHS/SOL (Hispanic Community Health Study/Study of Latinos) study.

## Conference Theme: “Genes, Behavior, and Environment: Putting the Pieces Together”

Dr Ivor Benjamin, AHA President, kicked off the meeting with a statistics update on the cardiovascular health of the United States, emphasizing the importance of addressing social determinants of health in achieving further progress toward improved cardiovascular health.

The opening session, “Genes, Behavior, and Environment: Putting the Pieces Together,” featured Dr Eric Boerwinkle, Dr Leslie Lytle, and Dr Michael Jerrett, and focused on addressing cardiovascular health through genomics, lifestyle behaviors, and the environment, respectively. Dr Boerwinkle, a genetic epidemiologist at the University of Texas Health Science Center at Houston's Medical School and dean of the School of Public Health, presented a broad overview of methodological advances in cardiovascular genomics. Notably, he discussed how genetic risk scores could be integrated with clinical care, providing case study examples demonstrating the roles of genetic risk scores in modifying traditional cardiovascular disease risk factors. Dr Lytle, a nutrition and health behavior researcher at the University of North Carolina at Chapel Hill Gillings School of Global Public Health, emphasized the need for intervention studies to effectively change behaviors associated with cardiovascular health, highlighting the CATCH (Child and Adolescent Trial for Cardiovascular Health) as such an example.^2^ Dr Jerrett, an environmental health sciences researcher at the University of California in Los Angeles Fielding School of Public Health, introduced the audience to the concept of the “exposome” with research examples measuring time geography of the exposome using smartphone technology, highlighting challenges of using microsensor data, and the importance of temporospatial factors in measuring environmental exposures.

In her talk, the “Intersection of Omics and Population Health,” Dr Svati Shah of the Duke Molecular Physiology Institute, expanded the genetics discussion to omics, biomarkers, and their role in epidemiology, before opening up the stage to oral abstract presentations.

## Memorial Lectures and Special Sessions

The David Kritchevsky Memorial Lecture was established in 2007, honoring Dr Kritchevsky's contributions to our knowledge of the impact diet has on atherosclerosis (Box 2). This year's honoree was Dr Barbara Howard, and she began her talk by acknowledging Dr Kritchevsky's mentorship and promotion of women in science. In her lecture, Dr Howard summarized the Women's Health Initiative Dietary Modification trial results, finding that an eating pattern emphasizing reduced dietary fat intake was associated with lower low‐density lipoprotein cholesterol, blood pressure (BP), and insulin, without increasing triglycerides or reducing high‐density lipoprotein cholesterol.^3^

Box 2 **Keynote and Memorial Lectures**

**Table:** 

Opening Session: “Genes, Behavior, Environment: Putting the Pieces Together” “Genes”—Eric Boerwinkle, PhD “Health Behavior: An Essential Piece of the Puzzle”—Leslie Lytle, PhD “Toward a Time Geography of the Exposome: Using Advanced Methods and Sensors to Characterize Exposures for Epidemiologic Research”—Michael Jerrett, PhD
Intersection of Omics and Population Health—Svati H. Shah, MD, MS, MHS, FAHA
ASPC Annual Debate: “Genetic Risk Scores for Cardiovascular Assessment—Hope or Hype?” Pro: “Genetic Risk Scores Are the Future for Cardiovascular Screening and Risk Assessment”—Christie M. Ballantyne, MD, FAHA Con: “Genetic Risk Scores Are the Emperor's New Clothes—Traditional CVD risk factors and other tools are enough”—Erin D. Michos, MD, MHS, FACC, FAHA, FASE
Physical Activity Guidelines “Highlighting the New Physical Activity Guidelines for Americans”—Janet E. Fulton, PhD, FAHA “Putting the Guidelines into Practice and What Are the Research Needs”—William E. Kraus, MD, FCC, FACSM, FAHA
The David Kritchevsky Memorial Lecture “A Low Fat Dietary Pattern and Health Outcomes—A Summary of the Women's Health Initiative 8 Year Trial and 18 Years of Follow‐up”—Barbara V. Howard, PhD, FAHA
The William B. Kannel Memorial Lecture “Fundamental Concepts of CVD Risk”—Vasan S. Ramachandran, MD, DM, FACC, FAHA
Special Lecture: “Implementing the NHLBI Strategic Vision in the Division of Cardiovascular Sciences”—David Goff, MD, PhD, FAHA

Dr David C. Goff, director of the National Heart, Lung, and Blood Institute Cardiovascular Sciences division, presented a special talk on “Implementing the NHLBI Strategic Vision in the Division of Cardiovascular Sciences.”^4^ The 6 focus areas are (1) address social determinants of cardiovascular health and health inequities, (2) enhance resilience, (3) promote cardiovascular health and prevent cardiovascular diseases across the lifespan, (4) eliminate hypertension‐related cardiovascular diseases, (5) reduce the burden of heart failure, and (6) prevent vascular dementia. These goals mirror the cardiovascular health determinants and cardiovascular disease risk factors highlighted throughout the AHA EPI|Lifestyle conference this year.

Dr Janet E. Fulton, Branch Chief, Division of Nutrition, Physical Activity, and Obesity at the Centers for Disease Control and Prevention, presented an overview of the new 2018 Physical Activity Guidelines for Americans, which were unveiled at the AHA 2018 Scientific Sessions in Chicago, Illinois.^5^ She highlighted the role of the guidelines advisory committee in evaluating the science to formulate the recommendations, and the key step of translating the Committee's Scientific Report^6^ into population guidelines and federal program implementations, including the CDC's Active People, Healthy Nation Initiative.^7^ New additions include physical activity guidelines for preschool‐aged children, and for adults, encouraging less sitting and more movement throughout the day; evidence for the benefits of increased movement include decreased anxiety and improved bone health, as well as better sleep quality.

Dr William Kraus, Professor at the Duke University School of Medicine, spoke about putting the new Physical Activity Guidelines in practice and highlighted the need for more research on the effects of different physical activity characteristics (eg, intensity) and types of activities on specific cardiovascular conditions. A key message repeated at AHA conferences over the past few years, and one that Dr Kraus echoed, is the importance of healthcare providers starting the conversation with patients about physical activity, including specifics on what type of activity to start with, addressing barriers to moving more, and helping patients identify and prioritize goals.

The William B. Kannel Memorial Lecture was established in 2013, honoring Dr Kannel's dedication and contribution to cardiovascular epidemiology. Dr Vasan S. Ramachandran has led the Framingham Heart Study for over 25 years—a study founded by Dr Kannel. Dr Ramachandran presented on fundamental risk factors of cardiovascular disease, highlighting landmark contributions over the past 60 years from biological to sociologic risk that develop and change over the lifecourse.

## Genes, Omics, and Biomarkers

A number of oral abstracts on metabolomics, proteomics, and biomarkers related to cardiovascular disease were presented at this year's conference. Dr Laura Corlin presented her research findings examining proteomics signature of cardiovascular risk in Framingham Heart Study participants, finding several proteomic signatures of cardiovascular disease risk factors. Steve Nguyen reported on the association between ideal adherence to the AHA Life's Simple 7 metric and epigenetic biomarkers of aging, finding that heart‐healthy lifestyles may slow biological aging among black adults. Alexander Razavi summarized the results of his study to identify metabolites associated with diastolic function in the Bogalusa Heart Study, finding evidence for subclinical disease markers in a biracial cohort of middle‐aged adults.

Dr Frank Qian presented his research finding on higher omega‐3 fatty acid biomarkers associated with lower risk of incident type 2 diabetes mellitus using participant‐level pooled data from 20 prospective cohort studies. Dr Yoriko Heianza discussed findings that revealed increased trimethylamine *N*‐oxide from gut microbiota was associated with elevated coronary heart disease risk in the Nurses’ Health Study, but this deleterious effect was mitigated by following a healthy dietary pattern. Using a novel marker of epigenetic aging called GrimAge,^8^ Dr Yinan Zhang showed how higher concentrations of triglycerides and lower concentrations of high‐density lipoprotein cholesterol were associated with more epigenetic aging, with some differences by sex and race. Dr Ming Ding discussed the association between several lipid‐based metabolites and habitual physical activity in the Nurses’ Health Studies and the Health Professionals Follow‐up Study participants, highlighting the opportunity to understand the biological mechanisms explaining the cardiovascular benefits of physical activity.

## Modifiable Risk Factors

### Physical Function, Physical Activity, and Nutrition

Dr John Bellettiere presented data from the OPACH (Objective Physical Activity and Cardiovascular Health) study^9^ indicating that poor lower extremity physical function was associated with increased cardiovascular disease risk and mortality among older women. Dr Andrea Z. LaCroix, also presenting data from the OPACH study, reported that time spent in daily life movement, derived from hip‐worn accelerometer and SenseCam data, was associated with reduced risk of cardiovascular disease, independent of traditional cardiovascular disease risk factors.

Dr John D. Omura presented his research on meeting physical activity guidelines in different cardiovascular disease risk groups, finding a lower proportion of those with existing cardiovascular disease met physical activity guidelines than those without cardiovascular disease, highlighting the need to promote physical activity to patients with cardiovascular disease. Dr Amanda Paluch presented her research findings that physical activity during middle age was associated with longer cardiovascular disease–free survival, with larger differences among women. Dr Sadiya S. Khan summarized her research on adherence to AHA physical activity guidelines and lower risk of heart failure, particularly among black men. She also presented research findings on diet pattern quality, with lower‐quality diets associated with twice the lifetime risk of heart failure compared with the highest‐quality diet pattern.

### Lifestyle Behavior Interventions

Dr Gail L. Daumit presented findings from the IDEAL (Intervention Trial to Decrease Cardiovascular Risk in Persons with Serious Mental Illness), an 18‐month intervention aimed at reducing cardiovascular risk among individuals with serious mental health illness. The active intervention (risk factor counseling and care coordination) was associated with a 10% difference in Framingham Risk Score compared with usual support (group exercise classes and access to healthy meals). Dr Huilin Tang discussed results from a network meta‐analysis of randomized control trials that found weight loss drugs were most effective in preventing type 2 diabetes mellitus compared with α‐glucosidase inhibitors, insulin sensitizers, and lifestyle modification interventions.

### Sleep

Dr Tianyi Huang reported on the association between actigraphy‐measured sleep regularity and risk of cardiovascular disease, where sleep irregularity was associated with higher incident cardiovascular disease in the MESA study. Dr Kelsie Full presented her research using isotemporal substitution models to assess potential cardiometabolic benefits associated with reallocating daily time spent in daytime activity, physical activity, and sleep. Among women with short sleep duration, replacing sedentary time with sleep was associated with improved cardiometabolic risk factors. Among women with longer sleep duration, reallocating time from sleep to physical activity provided benefit.

## The Built Environment and Social Determinants of Health

### Neighborhood Characteristics and Housing

Dr Jeong Hwan Kim presented his findings on the association between better neighborhood characteristics in the greater Atlanta area and a higher likelihood of AHA Ideal Life's Simple 7 score. Dr Justin Rodgers reporting that greater housing cost burden was associated with increased risk of hypertension, obesity, and depression, but not diabetes mellitus in adulthood, with stronger associations for males and renters. Dr Nrupen Bhavsar explained his research examining the impact of gentrification in Durham, North Carolina on increased hypertension and obesity prevalence among black long‐term residents compared with those who moved in or out of neighborhoods.

Dr Benjamin King, this year's winner of the Award for Excellence in Research Addressing Cardiovascular Health Inequities, showed that the prevalence of cardiovascular disease among the homeless population in Austin, Texas exceeds 20%. Among this population, cardiovascular disease was associated with other comorbid conditions and important barriers to health care, such as violence and coercion. Dr Kara M. Whitaker reported on her research examining neighborhood cohesion and physical activity. Better neighborhood characteristics were associated with increased physical activity in cross‐sectional analyses, but not 10‐year changes.

### The Built Environment and Cardiovascular Disease

Dr Yuk‐Lam Ho explained her research on mapping American College of Cardiology/AHA atherosclerotic cardiovascular disease risk in US veteran outpatients, showing geographic variation in atherosclerotic cardiovascular disease risk prediction versus atherosclerotic cardiovascular disease events, with notable overprediction in geographic areas closer to Veteran Affairs clinical centers. Dr Marcia Pescador Jimenez reported on early life exposure to green areas and diabetes mellitus in early adolescence. Greenness exposure throughout childhood was not associated with insulin resistance in early adolescence. Mingyu Zhang compared age‐related change in arterial stiffness in 2 tribes of hunter‐gatherers compared with Westernized high‐income populations, finding no notable differences across groups. Dr Kunihiro Matsushita presented findings on peripheral artery disease. In longitudinal National Health and Nutrition Examination Survey data, those with peripheral artery disease and coronary heart disease or stroke had worse survival than those with peripheral artery disease or coronary artery disease or stroke alone.

### Socioeconomic Status and Poverty

Dr Marialaura Bonaccio presented her research on increased cumulative socioeconomic disadvantage in the Moli‐sani study associated with increased risk of heart failure and atrial fibrillation hospitalizations. Dr Emily D'Agostino described her research findings that regardless of the poverty measure used in analyses, high‐poverty middle school students with poorer cardiovascular fitness had higher school absenteeism, with a particularly strong inverse association among girls attending schools with a large proportion of free/reduced‐price meals.

### Women's Health

Dr Muna J. Tahir presented findings from the CARDIA (Coronary Artery Risk Development in Young Adults) study that postmenopausal status was associated with reduced cerebrovascular reactivity, a clinical measure of cerebrovascular function and blood arrival. Saad Samargandy summarized his research looking at changes in abdominal visceral adipose tissue across pre‐, peri‐, and postmenopausal periods, finding a significantly faster increase in visceral adipose tissue the 2 years before menopause, a sensitive period for women's cardiometabolic health.

### Racial/Ethnic Disparities

Dr Caitlin Hicks highlighted racial disparities in adults undergoing carotid endarterectomy in the ARIC Study, with black participants less likely to undergo the procedure, even after adjustment for traditional risk factors and carotid intima‐media thickness. Dr Catherine Tcheandjieu reported that in the Million Veterans Program, polygenic risk scores had poorer performance for predicting coronary artery disease among black compared with European‐descent American veterans, highlighting the need to include non‐European descent populations in studies generating polygenic risk scores to improve clinical applicability.

Dr Jung‐Im Shin presented research on lower metformin use among blacks with diabetes mellitus because of the higher prevalence of impaired kidney function; however, following the eGFR‐based metformin label change, racial disparities in metformin use declined among those with moderately impaired kidney function.

## Primordial and Primary Prevention in Children and Adolescents

### Obesity and Physical Activity

Dr David Jacobs Jr presented preliminary findings from the International Childhood Cardiovascular Cohort (i3C) Consortium,^10^ where he found that childhood cardiovascular risk factors were associated with lower cardiovascular disease risk in middle‐to‐later adulthood than expected.

Dr Shakia Hardy presented her research suggesting that childhood obesity prevention efforts should focus on preventing excess weight gain before kindergarten and managing weight during summer breaks among school‐aged children. Dr Lindsay Pool modeled cardiovascular health trajectories among participants from 5 childhood/young adult cohorts in order to describe the timing of cardiovascular health decline and carotid intima‐media thickness. The childhood loss of 1 or more ideal cardiovascular health factors was associated with higher odds of developing subclinical atherosclerosis, measured by carotid intima‐media thickness compared with those who maintained ideal cardiovascular health.

### Smoking

Dr Tian Hu presented his research from the i3C Consortium finding that while cigarette smoking prevalence decreased from adolescence to adulthood, over 75% of those who smoked before age 12 years still smoked in their 20s, prompting a focus on strategies to prevent onset of early cigarette use in middle‐school‐age children.

## Hot Off the Press

The “Hot off the Press” session featured recent publications in *Journal of the American Medical Association*,* Circulation*, and *Annals of Internal Medicine*. Dr Jun Ma began the session with an overview of the RAINBOW (Research Aimed at Improving Both Mood and Weight) trial. The RAINBOW trial randomized 409 patients with obesity and depression to either a collaborative care intervention, which included a behavioral weight loss program with problem‐solving therapy and antidepressant medication if indicated, or usual care. Over the course of a year, patients in the collaborative care intervention experienced a greater decline in average body mass index and Depression Symptom Checklist score, compared with patients randomized to usual care; however, the effect sizes were modest.^11^

Dr Yuichiro Yano presented an analysis from the CARDIA study, using 4851 adults aged 18 to 30 years at enrollment in 1985. Study participants were categorized by the 2017 American College of Cardiology/AHA BP Guidelines based on the highest BP recording before the age of 40 years. Compared with young adults with normal BP, hazard of subsequent cardiovascular events increased incrementally for elevated BP, stage 1 hypertension, and stage 2 hypertension.^12^

Dr Tali Elfassy presented findings on income volatility during formative earning years (defined as 23–35 years of age) and adverse longitudinal outcomes in the CARDIA study. Income was assessed at 5 time points across 15 years (1990–2005), with income volatility quantified by the intra‐individual standard deviation for percent change in income, and “income drops” defined as ≥25% income decrease across the formative earning years. Both greater income volatility and frequency of income drops were associated with increased hazards of subsequent cardiovascular disease and all‐cause mortality.^13^

Dr Xiaoming Jia presented cost estimations for use of icosapent ethyl (highly purified eicosapentaenoic acid), following the REDUCE‐IT (Reduction of Cardiovascular Events with EPA‐Interventional Trial) results presented at AHA 2018 Scientific Sessions this past November. Dr Jia created a nationwide VA cohort comprising 263 144 patients with either established atherosclerotic cardiovascular disease or history of diabetes mellitus, the 2 criteria specified in the REDUCE‐IT. With federal discount pricing, the projected annual cost of treating these qualifying patients with icosapent ethyl would be $476 million.^14^

Dr William Applegate presented an analysis of intensive BP control and risk of dementia from the SPRINT MIND group, a secondary analysis of the SPRINT (Systolic Blood Pressure Intervention) trial, which randomized 9361 hypertensive adults >50 years of age to either a systolic BP goal of <120 (intensive) or <140 mm Hg.^15^ Randomization to intensive BP control did not significantly reduce risk of probable dementia, the primary outcome, but was associated with lower risk of composite mild cognitive impairment and/or probable dementia.

Dr Michael Blaha presented a descriptive analysis of e‐cigarette use in the United States among adults without a history of combustible cigarette smoking, based on data from the 2016 Behavioral Risk Factor Surveillance System. An estimated 1.9 million tobacco‐naïve Americans have begun using e‐cigarettes, with highest use among adults aged 18 to 24 years. E‐cigarette use varied substantially across states, and was associated with high‐risk lifestyle behaviors, binge drinking, and marijuana use.^16^

## American Society of Preventative Cardiology Annual Debate: Genetic Risk Scores for Cardiovascular Risk Assessment—Hope or Hype?

The American Society of Preventative Cardiology annual debate was held on the second‐to‐last day of the conference this year, increasing the attendance and the discussion. Dr Christie Ballantyne argued “pro” and Dr Erin Michos argued “con” for this debate on using genetic risk scores to assess cardiovascular risk. Trading points and jokes, Dr Ballantyne and Dr Michos put on a lively debate, engaging the audience and expert panel in a well‐informed discussion. Dr Ballantyne, on the “pro” side, likened traditional cardiovascular risk assessment to putting out fires—“too little too late”—versus early risk detection via genetic risk scores. He proposed universal screening for familial hypercholesterolemia and Lp(a) combined with polygenic risk scores for cardiometabolic diseases as 1 potential solution to this issue. Dr Michos, on the “con” side, presented 2 key questions on using genetic risk scores to guide clinical cardiovascular preventive therapy: will it change patient clinical management and will it change patient behavior? The majority of the time, she argued, genetic risk scores will not change clinical management—many preventive interventions will still be recommended regardless of genetic risk scores results, and other tools, such as coronary artery calcium testing, may provide more information regarding specific medication use such as statins, compared with genetic risk scores. This argument linked back to the opening session, when Dr Leslie Lytle noted studies showing that knowledge of increased genetic risk did not motivate patients to change their lifestyle behaviors, highlighting the need for effective translation of genetic risk to patients combined with effective interventions.^17^ Dr Ballantyne, Dr Michos, and the expert panel agreed that using genetic risk scores for cardiovascular disease risk assessment will require validation across diverse populations, comparison with relevant clinical models, and implementation studies to examine cost and reimbursement.

## Early Career Events

Following the annual EPI|Lifestyle Fun Run, 5 early career investigators competed in the 3‐Minute Rapid Fire Oral Abstract Competition, demonstrating concise science communication skills (Box 3). Katie Arlinghaus, the winner, presented her work “The Contribution of Intervention Frequency to Mexican American Adolescents’ Response to School‐based Obesity Intervention.” Dr Dale Mantey, runner up, presented his work on “Evaluation of the First Evidence‐Based E‐Cigarette Prevention Intervention: A Pilot Study.” The competition closed out with a keynote presentation from Dr Erin Michos, who spoke about her career journey and the role of the AHA in her success. She encouraged the audience to “keep a few pans in the fire,” to explore different research ideas related to your overall interest, to create a system of sponsors and mentors for support, and to keep showing up, no matter the number of grant and manuscript rejections.

Box 3 **Early Career 3‐Minute Rapid Fire Oral Abstract Competition Presented by the Council on Lifestyle and Cardiometabolic Health**

**Table:** 

Sajeevika Saumali Daundasekara, PhD	Influences of Early Childhood Economic Hardship on Adolescent Obesity
Katie Arlinghaus, MS (winner)	The Contribution of Intervention Frequency to Mexican American Adolescents’ Response to School‐Based Obesity Intervention
Fang‐Yu Lin, MS	The Potential Harms of Low Cholesterol Among Asians: Evidence from a Large Prospective Cohort
Dale Mantey, PhD (runner up)	Evaluation of the First Evidence‐Based E‐Cigarette Prevention Intervention: A Pilot Study
Chisom Odoh, PhD	Sex as a Predictor of Overnight and Emergency Treatment Among Homeless Adults

This year's Connection Corners were well attended, inspiring, and interactive, with clinicians, researchers, and teaching faculty participating. Dr Saverio Stranges shared key pieces of advice he wishes he had known as an early career investigator, including selecting research you are passionate about with a pragmatic lens regarding the likelihood of success, prioritizing a healthy work–life balance, and being brave in your career choices. Dr Stranges outlined a path to academic success that focused on team science and multidisciplinary research, maintaining a global perspective, and an eye toward diverse funding opportunities. Dr Alain Bertoni discussed conflicts of interest for early career researchers, detailing the different type of financial relationships investigators may have with drug companies. When considering consulting opportunities, Dr Bertoni encouraged the audience to choose research compensation as an informal grant over personal compensation. In the last session, Dr April Carson shared her advice on balancing research and teaching responsibilities as early career faculty. She emphasized deciding what your priorities are in terms of career, family, health, and hobbies and using the job interview as an opportunity to ask questions and learn about an institution's culture.

At the Lifestyle and Cardiometabolic Networking Luncheon, panelists discussed how to formulate relevant research questions that have an impact. Tips included reading relevant papers frequently and talking with colleagues to obtain a diversity of perspectives.

At the Epidemiology and Prevention Networking Luncheon, panelists discussed building patient engagement in research. Dr Mark Pletcher highlighted the importance of face‐to‐face meetings when engaging patients in research and truly listening to their ideas. Teresa Levitch, a patient advocate, encouraged the researchers in the audience to engage patients in groups instead of singularly to get a variety of patient perspectives. Dr Jennifer Blumenthal‐Barby recommended using icon arrays and other visual tools outlined in the International Patient Decision Aid Standards for presenting results to study participants. Other practical tips for involving patients in research from design to findings include piloting interview guides and online portals with patient groups, discussing the logistics of implementing an intervention, and engaging participants on project‐specific websites and social media pages.

Throughout the conference, early career events emphasized concise communication both to scientists and the lay public with tips for using your science to tell a story, both to patients and when writing grants. When discussing treatment options and prognosis with patients, Teresa Levitch shared straightforward tips, such as: patients do not care about what the research shows, so use your research to tell a story with your patient at the center. In the Friday morning early career session, Dr Allison Webel and Bailey DeBarmore conducted an interactive session on science communication using social media. Dr Webel spoke about using blogging to communicate your science, for the benefit of future scientists and the public, and shared how to include science communication activities in your promotion packets. To get started with science blogging, Dr Webel mentioned several possibilities including starting your own blog, working with your professional organization, such as the AHA Early Career Blogging Program, and talking with your institution or journals to do video abstracts, blog posts, and podcasts. Bailey DeBarmore discussed tips for effectively communicating science on Twitter, such as including a punchline, link, and image as well as swapping scientific jargon for simpler words that will improve understanding in both the lay audience and other scientific disciplines. The session concluded with an interactive exercise, where participants practiced what they learned by constructing tweets for example abstracts, and received live feedback from presenters and other participants.

## Conclusion

The 2019 EPI|Lifestyle Scientific Sessions brought together cardiovascular researchers from all over the nation and the world through dynamic programming and presentations. Researchers from every level—graduate students, early career investigators, and senior influencers in the field—contributed to thoughtful scientific discussion and fulfilling networking sessions (Boxes 4 and 5).

Box 4 **Conference Awards**

**Table:** 

Council on Epidemiology and Prevention	
Jeremiah and Rose Stamler Research Award for New Investigators	
Kristine MaWhinney	Cardiovascular Health and Cognitive Decline in Older Adults: The Cardiovascular Health Study
Jun Li (winner)	Dietary Inflammatory Potential Is Associated with Cardiovascular Disease Risk in Two Large Prospective Cohort Studies of US Men and Women
Khansa Ahmad	County Poverty Disproportionately Affects Mortality in Heart Failure Compared to Coronary Heart Disease
Matthew Harris	Rural vs Urban Living and Incident Cognitive Impairment in the Reasons for Geographic and Racial Differences in Stroke Study (REGARDS)
Edward Duran	Directly Measured Triglyceride‐Rich Lipoprotein Cholesterol and Small Dense LDL Cholesterol Concentrations Associated with Incident Cardiovascular Disease: Prospective Data from the Women's Health Study
Sandra A. Daugherty Award for Excellence in Cardiovascular Disease or Hypertension Epidemiology	
Hui Hu (winner)	Geographic Disparities in Hypertensive Disorders of Pregnancy: An Environment‐Wide Association Study
Di Zhao	Plasma Cyclic Guanosine Monophosphate (cGMP) and Heart Failure with Preserved Ejection Fraction: The Atherosclerosis Risk in Communities (ARIC) Study
Shilpa Bhupathiraju	A Healthy Plant‐Based Diet Index Is Favorably Associated with Cardiometabolic Risk Factors in the Mediators of Atherosclerosis in South Asians Living in America (MASALA) Study
Bamba Gaye	Temporal Trends of Cardiovascular Health Metrics and Population Attributable Risk for Mortality Among 366 270 French Adults
Roger R. Williams Memorial Award for Genetic Epidemiology and the Prevention and Treatment of Atherosclerosis	
Yoriko Heianza	Genetic Predisposition to Obesity and Healthful Plant‐Based Diet in the Risks of Hypertension and Cardiovascular Disease: Gene‐Diet Interaction Analysis in the UK Biobank
Trudy Bush Fellowship for CVD Research in Women's Health	
Tianyi Huang	Actigraphy‐Measured Sleep Regularity and Risk of Incident Cardiovascular Disease: The Multi‐Ethnic Study of Atherosclerosis
Guo‐Chong Chen	Body Fat and Cardiovascular Disease Risk in Postmenopausal Women With Normal Body Mass Index: the Women's Health Initiative
Jennifer Stuart	The Role of Aspirin in the Relationship Between Hypertensive Disorders of Pregnancy and Incident Maternal Cardiovascular Disease
Early Career Travel Award Winners	
Michael G. Nanna	
Kathryn E. Foti	
Bailey M. DeBarmore	
Tyler Quinn	
Jung‐Im Shin	
Nour Makarem	
Minority Travel Grant Winners	
Kendra D. Sims	
Sparkle Springfield	
Cecilia Castro‐Diehl	
Amira O. Collison	
Leanna Dumeny	
Debora Kamin Mukaz	
Jewel Scott

Box 5 **Conference Awards**

**Table:** 

Council on Lifestyle and Cardiometabolic Health	
Award for Excellence in Research Addressing Cardiovascular Health Equity	
Benjamin King	Heart Disease and Arrhythmia in a Sample Experiencing Homelessness: Epidemiology, Prior History and Risk Factors
Early Investigator Travel Award	
Andrea Lobene	Estimating Sodium Intake Using Timed Urine Collections from a Controlled Feeding Study
Laura Corlin	Proteomic Signatures of Cardiovascular Risk Factors: A Cross‐sectional Analysis of the Plasma Proteome in the Framingham Heart Study
Steve Nguyen	Adherence to Ideal Life's Simple 7 Metrics Is Associated with Epigenetic Biomarkers of Aging in African Americans: The Atherosclerosis Risk in Communities (ARIC) Study
Mingyu Zhang	Age‐Associated Rise in Arterial Stiffness Among Hunter‐Gatherers
Frank Qian	Omega‐3 Fatty Acid Biomarkers and Incident Type 2 Diabetes: An Individual Participant‐level Pooling Project of 20 Prospective Cohort Studies
Mark Bieber Award	
Sadiya Khan	Association of Dietary Patterns and Lifetime Risk of Heart Failure: The Cardiovascular Disease Lifetime Risk Pooling Project
Steven N. Blair Award for Excellence in Physical Activity Research	
Joowon Lee	Higher Fitness Level and Favorable Hemodynamic Responses to Submaximal Exercise Test in Young Adults Are Associated with Lower Risk of Chronic Kidney Disease in Later Life: The Framingham Study
Scott Grundy Fellowship for Excellence in Metabolism Research	
Brian Joyce	A Novel Epigenetic Link Between Gestational Diabetes Mellitus and Macrosomia
Shakia Hardy	Contemporary Estimates of Obesity Incidence Among Children in the United States
Kasra Moazzami	Combination of Obesity and Metabolic Syndrome Is Associated with Highest Rate of Depression Secondary to Increased Inflammation

Roger R. Williams Memorial Award funded by Merck, Inc. Award for Excellence in Research Addressing Cardiovascular Health Equity supported by the AHA Industry Nutrition Advisory Panel and donation by John M. Jackicic, PhD, FAHA, winner of 2018 Steven Blair Award. Additional awardee supported by donation from Emelia J. Benjamin, MD, ScM, FAHA, 2018 William B. Kannel Memorial Lecturer.

We look forward to the next year's AHA EPI¦Lifestyle Scientific Sessions, held March 3–6, 2020 in Phoenix, Arizona.

## Disclosures

Anderson is supported by NIH training grant T32‐HL007779. Delker is supported by NIH training grant 5‐T32‐HL079891‐12. Foti is supported by NIH training grant T32‐HL007024. Nanna is supported by NIH training grant T32‐HL069749‐15. Razavi is supported by NIH training grant 5TL1TR001418‐04. Scott is supported by the Robert Wood Johnson Foundation Future of Nursing Scholars program. Thomas is supported by NIH training grant T32‐HL007055. Turkson‐Ocran is supported by NIH training grant T32‐DK062707. The remaining authors have no disclosures to report.

## Authors’ Affiliations

From the Department of Epidemiology, Rollins School of Public Health, Emory University, Atlanta, GA (A.A.); Division of Epidemiology and Community Health, University of Minnesota, School of Public Health, Minneapolis, MN (M.D.A.); Division of Public Health Sciences, Department of Epidemiology & Prevention, Wake Forest School of Medicine, Winston‐Salem, NC (M.P.B.); Department of Cardiovascular Diseases, Mayo Clinic, Rochester, MN (S.‐A.B.); Division of Cardiology, University of North Carolina, Chapel Hill, NC (M.C.C.); Kidney Health Research Institute, Geisinger Health System, Danville, PA (A.R.C.); Joint Doctoral Program Public Health Epidemiology, San Diego State University and University of California at San Diego, San Diego, CA (E.D.); Department of Epidemiology, Johns Hopkins Bloomberg School of Public Health, Baltimore, MD (K.F., E.S.); Division of Chronic Disease Research Across the Lifecourse, Department of Population Medicine, Harvard Medical School and Harvard Pilgrim Health Care Institute, Boston, MA (V.G.); Duke Clinical Research Institute, Durham, NC (M.G.N.); Division of Cardiology, Duke University School of Medicine, Durham, NC (M.G.N.); Department of Medicine, Tulane University School of Medicine, New Orleans, LA (A.C.R.); Department of Epidemiology, Tulane University School of Public Health and Tropical Medicine, New Orleans, LA (A.C.R.); Duke University School of Nursing, Durham, NC (J.S.); Welch Center for Prevention, Epidemiology, and Clinical Research, Johns Hopkins University, Baltimore, MD (E.S.); Division of General Internal Medicine, Department of Medicine, Johns Hopkins School of Medicine, Baltimore, MD (E.S.); Department of Pediatric Cardiology, Stanford Cardiovascular Institute, Stanford School of Medicine, Stanford, CA (C.T.); Department of Epidemiology, Gillings School of Global Public Health, University of North Carolina at Chapel Hill, NC (A.G.T., B.M.D.); Johns Hopkins University School of Nursing, Baltimore, MD (R.‐A.N.T.‐O.); Frances Payne Bolton School of Nursing, Case Western Reserve University, Cleveland, OH (A.W.); Department of Research and Evaluation, Kaiser Permanente Southern California, Pasadena, CA (D.R.Y.).
